# Editorial: (Micro)Plastics and the environment

**DOI:** 10.1186/s12302-014-0016-3

**Published:** 2014-06-28

**Authors:** Martin Wagner, Magnus Engwall, Henner Hollert

**Affiliations:** 1 Department Aquatic Ecotoxicology, Goethe University Frankfurt, Max-von-Laue-Str. 13, Frankfurt, 60439 Germany; 2 MTM Research Centre, School of Science and Technology, Örebro University, Örebro, 70182 Sweden; 3 Department of Ecosystem Analysis, Institute for Environmental Research, ABBt - Aachen Biology & Biotechnology, RWTH Aachen University, Worringerweg 1, Aachen, 52074 Germany

**Keywords:** Plastic, Microplastics, Toxicity, Abundance, Aquatic environment, Risk assessment

## Abstract

(Micro)Plastics in the aquatic environment are an issue of emerging concern. However, to date, there is considerable lack of knowledge on the abundance and toxicity of plastic debris in aquatic ecosystems, especially with regard to the freshwater situation. In this editorial, we briefly discuss important aspects of the research on environmental (micro)plastics to stimulate research and call for papers.

*There are plastics in your toaster, in the blender and the clock, in the lamp and in the roaster, on the door and in the lock, in the washer and the dryer and the garden tools you lend, in your music amplifier and electric fryer—you have got a plastic friend!*

(DuPont advertisement show [1]).

Let us be honest: Humans love plastics. Parents learn about this when they watch their kids neglecting their beautiful, eco-friendly wooden toy train in favor of a garishly colored plastic truck made in China. But our affection with plastics runs much deeper than toy choice. With the invention of polymers, mankind transcended the limitations of natural materials regarding both their availability and properties [2]. One example: In 1867, *The New York Times* worried about the extinction of elephants, which were hunted for ivory, mainly needed to make billiard balls. The stagnant supply of ivory, so the story goes, fueled the search for faux ivory and finally led to the invention of celluloid in 1869. While this plastic (the term was not yet coined) was not particularly good for making billiard balls, its invention can be considered the dawn of the ‘plastic age’.

This is one story Susan Freinkel tells in her excellent book *Plastic – A Toxic Love Story* [3]. She uses eight plastic artifacts - comb, chair, Frisbee, IV bag, lighter, grocery bag, soda bottle, and credit card - to illustrate how plastics ‘built the modern world’ and how extremely close our relation to plastics is. However, besides all the fascination and benefits, our plastic love story becomes increasingly poisoned. Scientific reports on polymers releasing toxic chemicals have shaken the consumers’ trust in the safety of plastics. The most notorious plastic compound - bisphenol A - continues to provoke a riotous battle over its safety in the scientific, political, and public arena. Humans are ubiquitously exposed to this compound (a large US study found it in >90% of the participants, [4]), and piles of scientific studies demonstrate that it interferes with our hormonal systems. However, thanks to its economic value (and the respective lobbying efforts), bisphenol A is far from being regulated in many countries. Nonetheless, scientists implicate an increasing number of plastic-associated chemicals with adverse health effects in humans and in wildlife. This raises questions on whether we should critically revisit our relationship to plastics.

Another matter of concern is the accumulation of plastic debris in the environment. The use of plastics has increased almost 20-fold during the last 60 years, with annual production being 280 million tons 2011 [5]. An inevitable consequence has been a dramatic increase in the environmental release of plastic items due to absent recycling programs, negligence, and accidents. A business-as-usual scenario with continuing increase in plastics consumption predicts that in 2025, around 220 million tons of plastics will be discarded annually [6,7]. Much of this plastic debris will end up in the aquatic ecosystems, where the half-life of the polymers can range from months to centuries. A critical process is the fragmentation of larger debris to microplastics that occurs under environmental conditions (radiation, abrasion). Microplastics are defined as plastic pieces smaller than 5 mm in size. They are generated via degradation but also as primary microplastics, such a plastic pellets, which are used as raw material in the plastics industry.

The impacts of environmental plastics on the ecosystem are manifold, although our knowledge is still very fragmentary. Most attention is attributed to the detrimental effects of large plastic items on marine vertebrates. Often, birds, dolphins, fish, and turtles misinterpret plastic debris as prey. Extensive ingestion will result in obstruction and malfunction of the digestive tract. Likewise, animals can become entangled in plastics (e.g., in so-called ghost nets). Both events will cause starvation and eventually mortality [8]. Other effects of plastics are less visible; the ingestion of microplastics has been documented for a range of marine and freshwater species. In addition, there are reports that microplastics will accumulate along the food web. However, the biological effects of microplastics remain largely unstudied (see [9] for details).

Besides the direct effects of (micro)plastics, they may have an indirect impact on the ecosystem and on biota. Environmental plastics can act as vectors transporting invasive species and chemical pollutants. Regarding the former, organisms ‘hitchhiking’ on large plastic items may enter new habitats [8]. The same might be the case for microorganisms forming biofilms on (micro)plastics. Here, the so-called plastispheres will be inhabited by microbial communities different from the surrounding, including pathogens [10]. Regarding the latter, plastics contain and accumulate a complex mixture of anthropogenic pollutants. New evidence indicates that the equilibrium for a sorption of PAHs and PCBs to microplastics takes place within 1 year in the water [11]. There are some studies regarding the transfer of microplastics-associated chemicals to fish [6]. However, knowledge of how the ecosystems will be affected by plastics functioning as a vector for other pollutants is scarce. The same holds true for the composition and concentration of chemicals sorbing to (micro)plastics. Because of this lack of data, assessing the ecotoxicological risks is currently very difficult.

*Environmental Sciences Europe* (ESEU) is launching a new paper series entitled ‘(Micro)Plastics and the Environment’ to collate views and perspectives on the newest developments and insights into the environmental effects of plastics from different angles. The journal should be a bridge between scientists and all stakeholders, including the general public. We cordially invite all colleagues who feel they can contribute to the topic to submit a manuscript to ESEU with reference to this series.

Specifically we are interested in contributions to the following topics:

- Monitoring data for (micro)plastics in the aquatic environment
- Environmental chemistry of environmental (micro)plastics
- Exposure data of marine and freshwater species to microplastics
- Toxicity of environmental (micro)plastics to both marine and freshwater species
- Microbiology of environmental (micro)plastics
- Environmental (micro)plastics and European water strategies
- Risk assessment strategies for environmental (micro)plastics
- Strategies to mitigate the emission of (micro)plastics to the environment

## References

[1] ᅟ: *DuPont advertisement show entitled “The Wonderful World of Chemistry” at the 1964 New York World's Fair.* ᅟ: ᅟ; ᅟ. https://archive.org/details/TheWonderfulWorldOfChemistry-Dupont-1964NewYorkWorldsFair.

[2] Friedel RD (1983). Pioneer Plastic: the Making and Selling of Celluloid.

[3] Freinkel S (2011). Plastic: a Toxic Love Story.

[4] Calafat AM, Ye XY, Wong LY, Reidy JA, Needham LL (2008). Exposure of the US population to bisphenol A and 4-tertiary-octylphenol: 2003–2004. Environ Health Perspect.

[5] Wright SL, Thompson RC, Galloway TS (2013). The physical impacts of microplastics on marine organisms: a review. Environ Pollut.

[6] Rochman CM, Hoh E, Kurobe T, Teh SJ (2013). Ingested plastic transfers hazardous chemicals to fish and induces hepatic stress. Scientific Reports.

[7] Hoornweg D, Bhada-Tata P (2012). What a Waste: a Global Review of Solid Waste Management.

[8] Gregory MR (2009). Environmental implications of plastic debris in marine settings- entanglement, ingestion, smothering, hangers-on, hitch-hiking and alien invasions. Philos Trans R Soc Lond B Biol Sci.

[9] Wagner M, Scherer C, Alvarez-Muñoz D, Brennholt N, Bourrain X, Buchinger S, Fries E, Grosbois C, Klasmeier J, Marti T, Rodriguez-Mozaz S, Urbatzka R, Vethaak AD, Winther-Nielsen M, Reifferscheid G: **Microplastics in freshwater ecosystems: what we know and what we need to know.** *Environ Sci Eur* 2014, doi:10.1186/s12302-014-0012-7. 10.1186/s12302-014-0012-7 PMC5566174 28936382

[10] Zettler ER, Mincer TJ, Amaral-Zettler LA (2013). Life in the "Plastisphere": microbial communities on plastic marine debris. Environ Sci Technol.

[11] Rochman CM, Hoh E, Hentschel BT, Kaye S (2013). Long-term field measurement of sorption of organic contaminants to five types of plastic pellets: implications for plastic marine debris. Environ Sci Technol.

